# Alterations in the Expression of IFN Lambda, IFN Gamma and Toll-like Receptors in Severe COVID-19 Patients

**DOI:** 10.3390/microorganisms11030689

**Published:** 2023-03-08

**Authors:** Leonardo Sorrentino, Matteo Fracella, Federica Frasca, Alessandra D’Auria, Letizia Santinelli, Luca Maddaloni, Ginevra Bugani, Camilla Bitossi, Massimo Gentile, Giancarlo Ceccarelli, Ombretta Turriziani, Claudio Maria Mastroianni, Guido Antonelli, Gabriella d’Ettorre, Alessandra Pierangeli, Carolina Scagnolari

**Affiliations:** 1 Laboratory of Virology, Department of Molecular Medicine, Sapienza University of Rome, 00185 Rome, Italy; 2 Department of Public Health and Infectious Diseases, Sapienza University of Rome, Policlinico Umberto I of Rome, 00185 Rome, Italy; 3 Istituto Pasteur Italia, 00161 Rome, Italy

**Keywords:** interferon, COVID-19, immunology

## Abstract

Contradictory results have been reported regarding interferon (IFN) lambda (λ1–3) and IFN gamma (γ) production in COVID-19 patients. To gain insight into the roles played by these IFNs in SARS-CoV-2 infection, IFNλ1–3 and IFNγ mRNA expression was evaluated in peripheral blood mononuclear cells (PBMCs) (*n* = 32) and in cells of paired bronchoalveolar lavages (BALs) (*n* = 12). Lower IFNλ1–3 values (*p* < 0.001 for IFNλ1 and 3 and *p* = 0.013 for IFNλ2) in the PBMCs of severely ill patients were found compared to healthy donors (*n* = 15). Reduced levels of IFNγ were also detected in patients’ PBMCs (*p* < 0.01) and BALs (*p* = 0.041) compared to healthy donors. The presence of secondary bacterial infections was associated with decreased IFNλ amounts in PBMCs (*p* = 0.001, *p* = 0.015 and *p* = 0.003, respectively) but increased concentrations of IFNλ3 (*p* = 0.022) in BALs. Patients with alterations in C-reactive protein, lactate dehydrogenase and D-dimer levels had decreased IFNλ1 and 3 (*p* = 0.003 and *p* < 0.001) and increased IFNγ (*p* = 0.08) in PBMCs. Analyzing Toll-like receptors (TLRs) involved in IFN production, we found that TLR3 was highly expressed (*p* = 0.033) in patients with bacterial superinfections, while TLR7 and 8 (*p* = 0.029 and *p* = 0.049) were reduced in BALs of deceased patients. Overall, severe COVID-19 might be characterized by dysregulation in IFNγ, IFNλ and TLR3, 7 and 8 production.

## 1. Introduction

As a crucial component of innate immunity, interferons (IFNs) represent the first line of defense during the early phase of viral infection. Several studies have shown that the expression of IFNs is relevant in determining the outcome of SARS-CoV-2 infection, as it may change noticeably depending on age [1,2], gender [3], COVID-19 severity [4], the time points analyzed after SARS-CoV-2 infection [5], the type of clinical samples collected (i.e., upper/lower respiratory samples or blood) [6], the presence of anti-IFN alpha and omega neutralizing autoantibodies [7,8], and/or genetic defects in type I IFN-related encoding genes [9].

In addition, type III IFNs (IFNλs) have been shown to play a pivotal role during SARS-CoV-2 infection [10], and several studies have reported that type III IFNs show in vitro anti-SARS-CoV-2 effects [11,12]. Moreover, mucosal type III IFN response has been found to be impaired in severe COVID-19 patients [2,13], but other studies observed a detrimental role for type III IFNs in the lung repair process [14].

The antiviral action of type III IFNs is mainly observed in the mucosa, since the IFN lambda receptor (IFNLR) is primarily expressed in epithelial cells [15]. Nonetheless, their actions are also relevant in blood; for instance, during influenza A infection, IFNλs are mainly expressed in the endothelial tissues [15] and released during the early stages of viral infection. Subsequently, IFN gamma (IFNγ) production induces activation of natural killer (NK) cells and T lymphocytes [16,17,18]. Notably, a recent study found that IFNλ receptor activation in NK cells leads to IFNγ production [19], suggesting crosstalk between these types of IFNs. In agreement with this, a strong correlation has been found between type III IFN and IFNγ production during rhinovirus infection [20] and other diseases [21].

During SARS-CoV-2 infection, IFNλ expression is induced through the activation of retinoic acid-inducible gene-I (RIG-I)/melanoma differentiation-associated protein 5 (MDA5) (RIG-I/MDA5) [22], and IFNλ3 is stimulated by the Toll-like receptor (TLR)7–nuclear factor kappa B (NF-kB) pathway [23]. In SARS-CoV-2-infected patients, reduced levels of IFNλs in serum have been associated with COVID-19 severity [13]. On the other hand, excessive production of pro-inflammatory cytokines, including IFNγ, has been found to be associated with COVID-19 severity [24]. More recently, Zhang et al. showed that IFNγ together with TNF alpha stimulates macrophage expansion in lungs [25]. Moreover, IFNγ production is activated through pattern recognition receptor (PRR) pathways, including those associated with TLR4 [26] and IL-18/IL-12 signaling (antigen-nonspecific pathway) [27].

In light of these considerations, in order to provide additional insight concerning the expression of crosstalk between IFNλ and IFNγ production in COVID-19, we evaluated IFNλ and IFNγ gene expression in peripheral blood mononuclear cells (PBMCs) and bronchoalveolar lavage (BAL) cells collected from SARS-CoV-2-positive patients in terms of COVID-19 severity and outcome, the presence of bacterial superinfections and blood inflammatory marker levels. To further characterize IFN response in hospitalized SARS-CoV-2-infected patients, we examined the presence of anti-IFN neutralizing antibodies (NAB) and the expression of TLRs involved in IFN production and anti-SARS-CoV-2 response.

## 2. Materials and Methods

### 2.1. Patients and Clinical Sample Collection

Between March 2020 and April 2021, blood samples were collected from SARS-CoV-2-infected adult patients (*n* = 32) at the time of admission at Infectious Disease Unit, Policlinico Umberto I Hospital of Rome. All patients were unvaccinated against SARS-CoV-2; eight patients had fatal outcomes from SARS-CoV-2 infection. Paired BAL cells were obtained from 12 out of the 32 patients whose blood samples were collected. Blood samples from sex- and age-matched healthy donors (*n* = 15) were included in this study. Furthermore, BAL cells left over from routine diagnostic virological evaluations of age- and gender-matched SARS-CoV-2-negative patients were included. The local ethics committee approved the study protocol (Sapienza University of Rome, University Hospital “Policlinico Umberto I”, ref: 5836).

Plasma was separated for anti-IFN neutralizing antibody analysis and PBMCs were isolated using Lympholyte (Cedarlane, Burlington, Canada), lysed with TriZol (Zymo research, Irvine, CA, USA) and frozen at −80 °C for gene expression analysis. BALs were centrifuged at 13,000× *g* rpm; cell pellets were lysed with TriZol (Zymo research, USA) and frozen at −80 °C for gene expression analysis.

### 2.2. Gene Expression Quantification and Data Analysis

Total RNA from the cells of BALs samples and PBMCs was isolated using TriZol (Zymo research, USA) lysis buffer as previously described [28]. The reverse transcription was performed using a High-Capacity cDNA Kit (Applied Biosystems, Waltham, MA, USA). Gene transcript quantification was performed using TaqMan probes and a LightCycler 480 II (Roche, Basel, Switzerland). RT-PCR conditions, primers and probes for type III IFNs, IFNγ, TLR2, TLR3, TLR4, TLR7 and TLR8 have been previously described [28]. Accordingly, the difference in cycle thresholds (ΔCT) between β-glucuronidase (GUS) and the target genes was calculated for each gene in the sample. These values were converted to relative expression values using 2^−ΔCT^.

### 2.3. Anti-IFNγ Autoantibody Detection

Plasma samples collected from SARS-CoV-2-positive patients were assayed for neutralizing antibodies (NABs) against IFNγ (Sigma-Aldrich, USA) in a bioassay based on IFN-induced inhibition of the cytopathic effect of encephalomyocarditis (EMC) virus on human lung carcinoma epithelial cells (A549), as previously reported [7,8]. Briefly, twofold serial dilutions (starting from 1:10) of heat-inactivated plasma samples were incubated at 37 °C with 20 international units (IUs)/mL of the IFNγ preparation. After 1 h, the mixtures were added to duplicate monolayers of A549 cells (3 × 10^4^ cell/well) in 96-well microtiter plates. After 24 h, the cells were challenged with encephalomyocarditis virus (MOI = 0.05 TCID_50_/cell) and incubated at 37 °C for 24 h. Controls included a titration of the IFNγ preparation. Antiviral activity and its neutralization were assessed based on the virus-induced cytopathic effect. Cells were stained with crystal violet and the dye taken up by the cells was measured in a spectrophotometer at 570 nm. Titers were calculated using Kawade’s method, and the titers were expressed in tenfold reduction units (TRUs)/mL, where one TRU was the plasma dilution able to reduce the IFN titer from 10 to 1 IU/mL [29].

### 2.4. Statistical Analysis

Relative expression values were analyzed using the Mann–Whitney U test for pairwise comparison and Kruskal–Wallis test for multiple-group comparison. Correlation analyses were performed using Pearson’s correlation coefficient. The Pearson χ^2^ test was used for categorical variables. Statistical significance was set at *p* < 0.05. Statistical analyses were performed using SPSS v.27 and graphs were created using R software (2022.02.2 Build 485).

## 3. Results

### 3.1. Demographic and Clinical Characteristics of Patients

A total of 32 COVID-19 patients hospitalized at Policlinico Umberto I Hospital of Rome were enrolled in this study. Gender- and age-matched healthy donors (*n* = 15) were included.

The demographic and clinical characteristics of SARS-CoV-2-positive patients are shown in Table 1. The median days of hospitalization was 49.9. At the moment of hospitalization, 10 (32.3%) patients had >7000 neutrophils/mm^3^, whereas 28 (90.3%) had <1500 lymphocytes/mm^3^ and 9 (29.0%) had both >7000 neutrophils/mm^3^ and <1500 lymphocytes/mm^3^. Moreover, 27 patients (84.4%) had C-reactive protein (CRP) values > 1.5 mg/L and D-dimer values > 500 μg/L, while 19 (59.4%) had lactate dehydrogenase (LDH) > 300 U/L. Thirteen patients (40.6%) required admission to the intensive care unit (ICU). Moreover, three (9.4%) participants experienced thrombotic events, including ischemic and embolic events; four (12.5%) had bacterial pulmonary superinfections, including *S. pneumoniae, P. aeruginosa, A. baumannii* and *S. maltophila,* and three (9.4%) had bloodstream infections (BSIs), including *S. Hominis* and methicillin-resistant *S. Aureus* (MRSA). Lastly, eight (25.8%) patients had fatal outcomes of COVID-19.

### 3.2. Type III IFN Expression in PBMCs and BALs of COVID-19 Patients

Since IFNλ1–3 are produced early during respiratory viral infections [17,22] and type III IFN subtype levels have been reported to be differently expressed in respiratory and blood samples of severe COVID-19 patients [13,30], we stratified patients into those who required ICU admission and those with mild/moderate disease (Table 1). Afterwards, SARS-CoV-2 patients’ systemic and respiratory IFNλ1–3 transcript levels were compared with those measured in the PBMCs of healthy donors or BAL cells of SARS-CoV-2-negative patients (Table 1).

**Figure 1:**
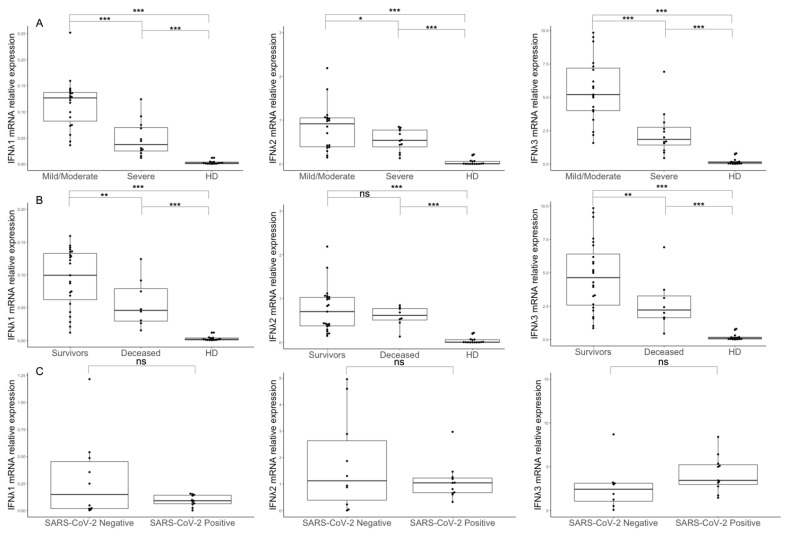
Type III IFN expression in the PBMCs and BALs of SARS-CoV-2-infected patients. IFNλ1, IFNλ2 and IFNλ3 mRNA levels measured in the PBMCs of COVID-19 patients with mild/moderate symptoms, patients with severe symptoms and healthy donors (**A**); in survivors, deceased patients and healthy donors (**B**); and in BAL cells of COVID-19 patients and SARS-CoV-2-negative patients (**C**). Statistical analyses were performed using the Mann–Whitney U test. *** *p* < 0.001, ** *p* < 0.01, * Interferon Response to SARS-CoV-2 Suggests a Role for*p* < 0.05, ns: not significant.

We found that transcript levels of IFNλ1, IFNλ2 and IFNλ3 were increased in both mild/moderate and severe COVID-19 patients compared to healthy donors (Figure 1A, *p* < 0.001 for all genes). However, we found reduced transcript levels for IFNλ1, IFNλ2 and IFNλ3 (*p* < 0.001 for IFNλ1 and 3 and *p* = 0.013 for IFNλ2) in PBMCs collected from critically ill patients compared to those observed in patients with mild/moderate disease (Figure 1A).

Lower blood expressions for IFNλ1 and 3 were also observed in patients with fatal COVID-19 outcomes compared to survivors (*p* = 0.004 for both genes, Figure 1B).

With regard to the analysis of type III IFN expression in BALs, we did not find any differences in the levels of IFNλs between SARS-CoV-2-infected and -negative patients (Figure 1C), nor when comparing patients stratified according to age, gender and disease severity. However, after dividing COVID-19 patients into those with (*n* = 4) or without pulmonary bacterial superinfections (*n* = 8), we found increased mRNA expression for IFNλ3 (*p* = 0.022) but not for IFNλ1 or 2 mRNA in BAL cells (Table 2). In contrast, lower expressions of IFNλ1, 2 and 3 (*p* = 0.001, *p* = 0.015 and *p* = 0.003, respectively) were found in the PBMCs of patients with pulmonary bacterial superinfections compared to negative ones (Table 2).

### 3.3. Type II IFN Expression in PBMCs and BALs of COVID-19 Patients

Since IFNγ production occurs later than that of the other types of IFNs during viral infections [19], and given the recently proposed crosstalk between IFNγ and IFNλs [20], we evaluated the IFNγ mRNA expression in PBMCs and BAL cells of SARS-CoV-2-infected patients, healthy donors (PBMCs) and SARS-CoV-2-negative patients (BALs) (Table 1).

**Figure 2:**
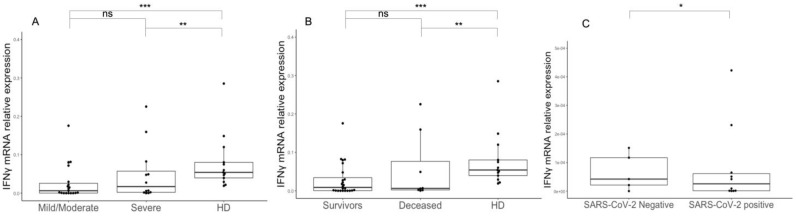
Type II IFN expression in PBMCs and BAL cells of SARS-CoV-2-infected patients. IFNγ mRNA levels were measured in PBMCs of COVID-19 patients with mild/moderate symptoms, patients with severe symptoms and healthy donors (**A**); in PBMCs of COVID-19 survivors, deceased patients and healthy donors (**B**); and in BAL cells of COVID-19 patients and SARS-CoV-2-negative patients (**C**). Statistical analyses were performed using the Mann–Whitney U test. *** *p* < 0.001, ** *p* < 0.01, * *p* < 0.05, ns: not significant. Transcript expression of IFNγ in cells collected from BALs showed the same trend as that observed in PBMCs, with lower gene expression in patients compared to SARS-CoV-2-negative ones (*p* = 0.041) (Figure 2C). In contrast, we did not observe any differences in IFNγ production in patients’ BAL cells and PBMCs depending on the presence of pulmonary bacterial superinfections. Lastly, there was a weak negative correlation between levels of IFNγ and those of IFNλ subtypes (IFNλ1, r = −0.307, *p* = 0.005; IFNλ2, r = −0.263, *p* = 0.018; and IFNλ3, r = −0.362, *p* < 0.001, respectively).

We found reduced expression of IFNγ in the PBMCs of patients with severe or mild/moderate disease compared to that in healthy individuals (*p* < 0.01) (Figure 2A). Furthermore, we observed decreased expression of IFNγ mRNA in the PBMCs of both survivors and deceased patients compared to healthy donors (*p* < 0.01, Figure 2B).

Since reduced type I IFN and ISG levels have been reported to be associated with the presence of neutralizing antibodies against IFN alpha/omega [7,31], and as we had observed decreased IFNγ expression in COVID-19 patients, we tested patients’ plasma for the presence of anti-IFNγ NAB activities, but all plasma samples showed negative results (NAB titer against IFNγ < 10 TRU/mL).

### 3.4. Type II and III IFN Expression Is Associated with Blood Inflammatory Markers

**Figure 3:**
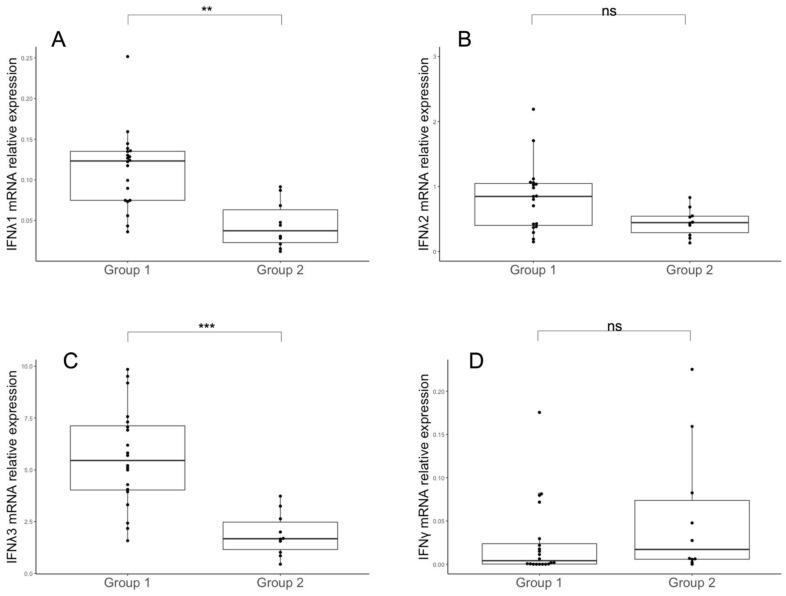
IFNλ and IFNγ expression in PBMCs according to levels of blood inflammatory markers. Levels of IFNλ1 (**A**), IFNλ2 (**B**), IFNλ3 (**C**) and IFNγ (**D**) mRNA in COVID-19 patients stratified into two groups (group 1: patients with one or two altered inflammatory markers (*n* = 15), group 2: patients with three inflammatory markers altered (*n* = 15)). Normal ranges considered for the analysis were the following: CRP < 1.5 mg/L, LDH > 80 and <300 U/L and D-dimer < 500 μg/L [32]. Statistical analyses were performed using the Mann–Whitney U test. *** *p* < 0.001, ** *p* < 0.1, ns: not significant.

As changes in blood biochemical parameters, such as CRP, LDH and D-dimer, have been associated with the severity of COVID-19 [32], we analyzed the expression of IFNλs and IFNγ in SARS-CoV-2-infected patients stratified in two groups: those who had one or two parameters with increased levels (*n* = 15, group 1) and those who had enhanced levels of all three parameters (*n* = 15, group 2). Only two patients had normal levels for the three parameters, while none showed decreased levels of CRP, LDH or D-dimer. We observed reduced mRNA expression for IFNλ1 and IFNλ3 (Figure 3A, *p* = 0.003, and 3C, *p* < 0.001, respectively) and a trend toward a reduction for IFNλ2 (Figure 3B, *p* = 0.08) in PBMCs from patients with altered levels of CRP, LDH and D-dimer. In contrast, there was an opposite trend for IFNγ (Figure 3D, *p* = 0.08), which moved toward upregulation in gene expression in patients with changed levels for the three inflammatory markers.

Since BAL cells were collected from severe COVID-19 patients, all the inflammatory parameters analyzed (*n* = 3) were profoundly changed [32]. Furthermore, we found a positive correlation between IFNγ expression and LDH levels (Spearman r = 0.682, *p* < 0.001, in BAL cells. We did not find a correlation between expressions of the other genes and the biochemical markers. 

### 3.5. TLR Expression in Lower Respiratory Tract of Severe Patients

**Figure 4:**
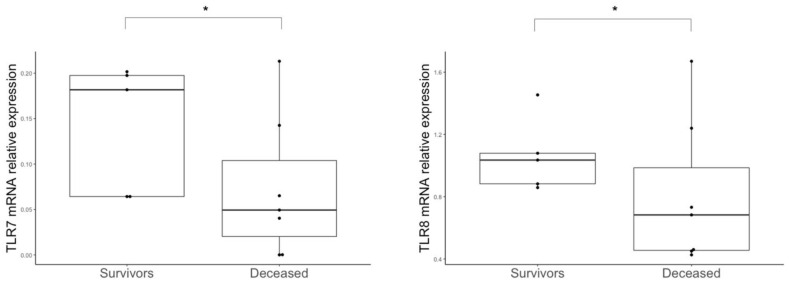
TLR expression in BALs. Levels of TLR7 and TLR8 mRNA in BAL cells of non-survivors (*n* = 8) and survivors of COVID-19 (*n* = 4). Statistical analyses were performed using the Mann–Whitney U test. * *p* < 0.05.

Having observed dysregulation in the levels of IFNλs and IFNγ in BAL cells from COVID-19 patients, and given that TLR2 [33], TLR3 [34], TLR4 [35] and TLR7/TLR8 [36] are involved in anti-SARS-CoV-2 response and related to IFN production after viral and/or bacterial infections [37], we investigated whether these TLRs were differently expressed in COVID-19 patients. We found that patients with fatal outcomes following SARS-COV-2 infection expressed decreased levels of TLR7 and TLR8 (*p* = 0.029 and *p* = 0.049, respectively) (Figure 4). In contrast, increased expression of TLR3 (*p* = 0.033) was observed in BAL cells of COVID-19 patients who had pulmonary bacterial superinfections (Table 2). There were no differences for the other TLRs analyzed (TLR2 and TLR4).

## 4. Discussion

Due to the contrasting results obtained so far, the expression of IFNλs in the lower respiratory tract and blood of COVID-19 patients still needs to be further characterized. SARS-CoV-2 proteins impair MDA5/RIG-I and TLR3-TRIF signaling, leading to suppression of type III IFNs [34,38]. At the same time, several *in vitro* studies have shown strong antiviral effects of type III IFNs against SARS-CoV-2 replication [39,40]. Furthermore, Sohn et al. recently showed that intranasal treatment with IFNλ decreased immunopathogenesis associated with SARS-CoV-2 in transgenic mice expressing angiotensin-converting enzyme II (ACE-II) [41]. The above findings provide evidence supporting the protective anti-SARS-CoV-2 action of these cytokines belonging to the IFN system.

In our study, there was an alteration in the transcript expression of IFNλs in BAL cells and PBMCs collected from severe SARS-CoV-2-infected patients, including those who experienced fatal outcomes. In agreement with these results, several studies have reported that IFNλ expression is downregulated in the upper airways of patients who developed severe symptoms during the early stage of SARS-CoV-2 infection [2,13], while another study reported that only the IFNλ2 subtype was upregulated in both the upper and lower respiratory tracts of critically ill patients [4]. Our research group previously observed a trend toward lower mRNA expression for type III IFNs in cells collected from the nasopharyngeal swabs of critically ill patients hospitalized during the first wave of the COVID-19 pandemic [2]. This scenario concerning a reduction in IFNλ levels in SARS-CoV-2-positive patients was confirmed during the first wave of the pandemic by Fukuda et al. [13]. Notably, we recently observed that endogenous levels of IFNλs were lower in upper respiratory samples from adults than in those from adolescents and children [1], indicating that the expression of type III IFNs differs depending on age [42] and highlighting the complexity of the immunological phenomenon analyzed.

Remarkably, patients with pulmonary bacterial superinfections expressed reduced levels of IFNλs in PBMCs but increased amounts of IFNλ3 mRNA in BALs cells, confirming previous findings showing that type III IFNs are produced during bacterial respiratory infections [43,44]. IFNλs might promote the maintenance of epithelial barrier integrity [45], and they were shown to have a protective role during *P. aeruginosa* pneumonia in a mice model [46]. In contrast, Broggi et al. observed increased lung damage associated with IFNλ induction upon viral recognition that could favor bacterial pulmonary superinfections [47].

With regard to the analysis of IFNγ levels in COVID-19 patients, transcript amounts for IFNγ were reduced in the lower respiratory tracts and PBMCs of critically ill patients compared to healthy donors despite all patients had negative results for plasma anti-IFNγ autoantibody detection. In agreement with these findings, a previous study showed that lower levels of IFNγ were associated with greater COVID-19 severity [48]. However, these results are in contrast with further previous findings, in which IFNγ protein levels in blood were upregulated in severe COVID-19 patients, contributing to a “cytokine storm” [24,49]. Possible explanations for this discrepancy are the following: i) a lower number of T lymphocytes expressing IFNγ have been reported in the blood of convalescent COVID-19 patients compared to healthy donors [50]; ii) increased frequencies of exhausted NK cells expressing lower levels of IFNγ have been described in COVID-19 patients [51]; iii) the antiviral transcriptional response in circulating immune cells has been shown to be strongly associated with a specific subset of IFNs, most prominently IFNα2 and IFNγ, and differential IFN subtype production was linked to distinct circulating immune cell types [52]. The above aspects were not measured in our study.

Furthermore, another variable could be the temporal collection of clinical samples from the onset of SARS-CoV-2 infection, which remains unstudied.

Remarkably, we observed a negative correlation between IFNγ and the three IFNλs in PBMCs. To the best of our knowledge, no other studies have described this correlation between type II and III IFNs during SARS-CoV-2 infection. In this context, it has been shown that NK cells express IFNλ receptor, and its activation leads to IFNγ production [19]. In support of the relationship between type II and III IFNs, we recorded a dysregulation in IFNγ and IFNλ gene expression levels in those patients with changed LDH, CRP and D-dimer values, which are known inflammatory markers associated with COVID-19 severity [32]. Moreover, IFNγ concentrations were positively correlated with LDH levels. Interestingly, several studies have found that IFNγ-induced protein 10 (IP-10), together with CRP, is a relevant marker of mortality in COVID-19 [53,54]. At the same time, reduced serum IFNλ2 levels have been associated with greater disease severity [55], suggesting low antiviral activity and SARS-CoV-2 persistence.

Having observed differential expression of type III and II IFNs in the lower respiratory tracts of COVID-19 patients, we evaluated TLR expression in BALs; in particular, deceased patients showed lower expression levels of TLR7 and TLR8 mRNAs. In this context, the TLR7/8 pathway has been reported to be involved in SARS-CoV-2 recognition and subsequent activation of IFN pathways [36]. Impaired expression of TLR7/8 has been previously observed in BALs of severe COVID-19 patients [56]. We found that levels of TLR3 mRNAs were increased in those patients with pulmonary bacterial superinfections. Few data are available concerning TLR3 levels in COVID-19 patients, but it has been shown that TLR3 activates type I/III IFNs through the TRIF factor [57]. Moreover, the presence of the single nucleotide variant (SNV) Rs3775291 in the TLR3 locus has been related to COVID-19 severity [58], highlighting the importance of this TLR in the immunological recognition of SARS-CoV-2. Despite TLR2 and TLR4 being involved in anti-SARS-CoV-2 immune response [33,35], we did not find any significant difference between SARS-CoV-2-positive and -negative patients’ BALs.

The limitations of this study were the small size of the sample of COVID-19 patients analyzed; the potential heterogeneity in SARS-CoV-2-negative patients’ BAL cells, in which IFNs and TLRs could have been activated due to the presence of other respiratory pathogens; and the lack of asymptomatic SARS-CoV-2-positive patients. Moreover, the analysis of IFN and TLR gene expression was performed in a period when the original strain and then the alpha variant of SARS-CoV-2 were predominant [59]. Thus, the lack of information about SARS-CoV-2 variants and their relationship with IFN/TLR expression represents another limitation. Moreover, we did not carry out an evaluation of the frequencies of the immune cellular subsets and their relationships with the expression levels of IFNs. A lack of data on protein levels represents another limitation of this study.

In conclusion, these findings indicate that patients with severe SARS-CoV-2 infection, including those characterized by increased levels of blood inflammatory markers and/or the presence of secondary bacterial superinfections and non-survivors of COVID-19, had altered expression of IFNλ subtypes, IFNγ and TLR3, TLR7 and TLR8. Further studies are urgently needed to better define the roles of IFN response and TLR pathways during SARS-CoV-2 infection by analyzing larger group of patients, including those with asymptomatic infections and those infected with the novel SARS-CoV-2 variants.

## Figures and Tables

**Table 1:** Demographic and clinical characteristics of SARS-CoV-2 patients, SARS-CoV-2-negative patients and healthy donors.

Features	Total Patients (*n* = 32)	Mild/Moderate Patients (*n* = 19)	ICU-Admitted Patients (*n* = 13)	*p*-Value	SARS-CoV-2-Negative Patients (*n* = 10)	*p*-Value **	Healthy Donors (*n* = 15)
Male (N (%))	27 (84.4)	18 (94.7)	9 (76.9)	>0.1	6 (60.0)	>0.1	10 (66.7)
Female (N (%))	5 (15.6)	1 (5.3)	4 (23.1)	>0.1	4 (40.0)	>0.1	5 (33.3)
Age (years)	72 (37–86)	72 (37–80)	72 (38–86)	>0.1	70 (36–87)	>0.1	68 (41–76)
NC (cell/mm^3^)	6734.85 (1920–18,000)	6543.68 (2100–14,440)	7249.23 (1920–18,000)	NA	4862 (2530–6930)	NA	NA
LC (cell/mm^3^)	903.13 (170–3890)	1128.42 (350–3890)	610.77 (170–1050)	NA	1297 (940–1960)	NA	NA
MC (cell/mm^3^)	380 (60–970)	411.58 (150–970)	348 (60–780)	NA	333 (190–440)	NA	NA
CRP (mg/L)	7.39 (0.1–30.9)	6.52 (0.1–21.2)	10.33 (1.9–30.9)	NA	14.43 (5.2–19.7)	NA	NA
Patients with normal ranges of CRP * (N (%))	5 (15.7)	5 (26.3)	0 (0.0)	<0.001	0 (0.0)	>0.1	NA
LDH (U/L)	354.69 (110–614)	299.89 (110–514)	445.5 (177–614)	NA	NA	NA	NA
Patients with normal ranges of LDH * (N (%))	13 (40.6)	10 (52.6)	3 (23.1)	>0.1	NA	NA	NA
D-dimer (μg/L)	1892.65 (197–4610)	1050.8 (197–4445)	3302.9 (1014–4610)	NA	NA	NA	NA
Patients with normal ranges of D-dimer * (N (%))	5 (15.6)	5 (26.3)	0 (0.0)	<0.001	NA	NA	NA
BSI (N (percentage))	3 (9.4)	0 (0.0)	3 (23.1)	>0.1	2 (20.0)	>0.1	NA
PSI (N (percentage))	4 (12.5)	0 (0.0)	4 (30.8)	>0.05	4 (40.0)	>0.1	NA
DP (N (percentage))	8 (25.0)	0 (0.0)	8 (61.5)	0.02	NA	NA	NA

Data are expressed as means (range). NC: neutrophil count, LC: lymphocyte count, MC: monocyte count, CRP: C-reactive protein, LDH: lactate dehydrogenase, BSI: bloodstream bacterial infection, PSI: pulmonary superinfection, ICU: intensive care unit, DP: deceased patients. * Normal ranges considered for CRP were <1.5 mg/L; for LDH, >80 and <300 U/L; and for D-dimer, <500 μg/L. ** These *p*-values were calculated by comparing SARS-CoV-2-positive patients admitted in the ICU and SARS-CoV-2-negative patients. Pulmonary superinfections in positive patients were related to *S. pneumoniae*, *P. aeruginosa*, *A. baumannii* and *S. maltophila*, while bacterial species found in bloodstream infections were S. *Hominis* and methicillin-resistant S. *Aureus* (MRSA). Bacteria found in SARS-CoV-2-negative patients with pulmonary infections were *K. oxytoca, H. influenzae, P. aeruginosa* and *E. cloaceae.*

**Table 2:** IFNλ and TLR gene expression in COVID-19 patients with and without secondary bacterial pulmonary infections.

	PBMC			BAL		
Gene	Positive *	Negative	*p*-Value	Positive *	Negative	*p*-Value
IFNλ1	0.03 (0.02–0.09)	0.09 (0.01–0.25)	0.001	0.11 (0.09–0.14)	0.08 (2.08 × 10^−3^–0.15)	>0.1
IFNλ2	0.38 (0.13–0.68)	0.78 (0.15–4.41)	0.015	2.01 (1.05–2.97)	0.81 (0.32–1.46)	>0.1
IFNλ3	1.33 (0.45–3.73)	4.17 (0.85–9.85)	0.003	6.74 (5.09–8.39)	3.29 (1.45–6.41)	0.022
TLR2	NA	NA	NA	0.81 (0.05–2.47)	0.44 (0.02–11.71)	>0.1
TLR3	NA	NA	NA	1.67 (1.56–1.79)	1.23 (0.6–1.56)	0.033
TLR4	NA	NA	NA	2.51 (1.85–3.61)	1.89 (1.06–8.28)	>0.1
TLR7	NA	NA	NA	0.07 (2.31 × 10^−4^–0.14)	0.06 (6.49 × 10^−5^–0.21)	>0.1
TLR8	NA	NA	NA	0.73 (0.68–1.67)	0.88 (0.42–1.45)	>0.1

Data are expressed as medians (range) of 2^−ΔCt^. Statistical analysis was performed using the Mann–Whitney U test. NA: not available. * Bacteria species found were the following: *S. pneumoniae*, *P. aeruginosa*, *A. baumannii* and *S. maltophila.*

## Data Availability

The datasets analysed during this study are available from the corresponding author on reasonable request.
